# The Impact of Microbiota on Musculoskeletal Injuries

**DOI:** 10.3390/cells14070554

**Published:** 2025-04-07

**Authors:** Giada La Placa, Marcello Covino, Marcello Candelli, Antonio Gasbarrini, Francesco Franceschi, Giuseppe Merra

**Affiliations:** 1 School of Specialization in Food Science, University of Rome Tor Vergata, 00133 Rome, Italy; laplacagiada@gmail.com; 2 PhD School of Applied Medical-Surgical Sciences, University of Rome Tor Vergata, Via Montpellier 1, 00133 Rome, Italy; 3 Department of Emergency Medicine, “A. Gemelli” General Hospital Foundation IRCCS, Catholic University of Sacred Heart, 00168 Rome, Italymarcello.candelli@policlinicogemelli.it (M.C.);; 4 Department of Medical and Surgical Sciences, “A. Gemelli” General Hospital Foundation IRCCS, Catholic University of Sacred Heart, 00168 Rome, Italy; 5 Department of Biomedicine and Prevention, University of Rome Tor Vergata, 00133 Rome, Italy

**Keywords:** musculoskeletal, microbiota, dysbiosis

## Abstract

Musculoskeletal injuries comprise a wide range of physical conditions impacting the coordination of bones, muscles, and joints. Estimations suggest that close to one-third of the world’s population will experience a musculoskeletal or non-musculoskeletal injury at some point in their life. Musculoskeletal injuries affect athletes, office workers, industrial workers, older adults, and children every year. Among individuals over the age of 65, musculoskeletal injuries disproportionately affect older women, limiting their ability to maintain an active and professional life or engage in leisure activities during retirement. The field of physical therapy has recently expanded to build an understanding of the complex, non-linear interactions between the gut microbiota and the musculoskeletal system. There is an unexpected connection between the gut microbiota and both the experience of musculoskeletal pain and the healing process following musculoskeletal injuries. Understanding the mechanisms of the microbiota’s influence on these injuries could inform healthcare strategies aimed at prevention and recovery. For patients who suffer from or are at risk of developing musculoskeletal injuries, analyzing the composition of their microbiota plays a crucial role in patient stratification, which can significantly enhance the effectiveness of prevention and treatment strategies.

## 1. Introduction to Musculoskeletal Injuries

Microbiota shape multiple systems of the human body in ways that may contribute to healthcare maintenance or disease pathology, including the musculoskeletal system. The gut microbiota interacts in a complex manner with homeostasis, also involving the nervous and immune systems. How can these complex connections influence the musculoskeletal system’s physiology and pathology? To answer this question, we have to observe the gut microbiota as an organization comparable to the stem cell niches of musculoskeletal microenvironments, sharing overlapping cytokines and metabolites. An understanding of these varieties of microbiota and muscle/skeletal system crosstalk may prompt clinical and regenerative strategies that steer musculoskeletal health by utilizing novel “cell” types that are outside of tissues, in a “non-canonical” stem cell niche [1]. The term microbiota is a “plural tantum” term for all organisms living in or on another multicellular organism, which is defined as the microbiota’s host. Bacteria outnumber human cells with a ratio of 1.3:1; hence, as their immunological and developmental relationships with mammals are extensive and enduring, the host’s organs and the mechanisms involved in these processes have evolved multiple times. Inspiratory microorganisms occupy numerous ecological niches, including large areas of the gut, which are never internally connected. Other surfaces like the skin and the eyes are similarly colonized, even though they do not have the health relevance that the gut has. These microbes are necessary for wellness preservation and are also linked to different host systems and disorder progression. Here, we explore how the microbiota are responsible for gastrointestinal system activity and can influence injury states, with a view on how to fill the gap in the literature and support the development of new clinical strategies [2,3,4,5]. 

### 1.1. Definition and Role of Microbiota

The term microbiota refers to the microorganisms inhabiting on, in, and around the body, tightly associated with the host. Most of these commensals are bacteria and other organisms such as fungi, viruses, and archaea, which make up the larger microbial community [2]. The bacteria within individuals, whether commensal or pathogenic, are diverse and inhabit various body sites, consisting of unique microenvironments [6,7]. Bacteria continuously cooperate in extensive metabolic processes with various members of the body’s homeostasis, which is more complex than previously appreciated. Commensal bacteria on mucosal surfaces and the skin heavily regulate the immune system by acting as antigens or adjuvants, activating various signaling pathways, or consuming the nutrients that pathogens require, inhibiting their growth [8]. Various studies state that changes in the microbiota composition are related to overall health. The gut microbiota in adults is actively engaged in health or diseases through the contribution of bacterial genes to the overall gene pool. Pathogenesis and infection generally result from a perturbation of the body’s homeostasis, favoring the propagation of an increasingly pathogenic microbial community. Despite the high level of interpersonal variability in the microbiota composition of humans, the amount of operational taxonomic units is relatively constant between individuals. Several familial factors, such as diet, significantly influence the diversity of the microbiota, with further environmental, host, or accidental factors possibly shaping the bacterial communities within individuals. Dysbiosis within the microbiota, defined as a change in the composition of these microorganisms, is frequently associated with disease onset, complications, progression, or recovery. Musculoskeletal diseases, including complex forms such as pain, are not merely physical imbalances. A substantial inflammatory process, of either endogenous or exogenous origin, usually coexists or precedes further complications sustaining or exacerbating the initial complaint. Broad-spectrum antibiotic treatments significantly alter gut microbiota composition. They produce major transformations within the microbial community, but the microbiota rebound fairly quickly after the interruption of treatment [9].

### 1.2. Anatomy and Function of the Musculoskeletal System

The musculoskeletal system is responsible for providing structure to the body and protecting the body from internal and external harmful factors. It consists of bones, muscles, tendons, and ligaments [2]. Bones are the primary component, forming the structure and providing support to the body. They are connected by ligaments, which are fibrous tissues which attach bone to bone. Muscles are connected to bones by tendons, fibrous tissues which link muscles to bone. Muscles act upon the bones to produce movement. Movement is controlled by the nervous system, which is connected to muscles by the neuromuscular system. The musculoskeletal system has many vital functions in the body, including support, movement, protection of internal organs, mineral homeostasis, and hematopoiesis. The musculoskeletal system works smoothly and seamlessly through collaborative activities of its components. Muscles provide force to pull on the bones, which act as a lever system to produce a movement. Tendons that attach the muscles to the bone are used efficiently to conduct the force, act as a pulley system, and keep the muscle’s equilibrium. In the meantime, ligaments attach bone to bone and keep the joint stable. This is an example of how a complex activity like walking can be perfectly performed through collaboration of the bones/muscles and neuromuscular system. An understanding of how the musculoskeletal system works to maintain body mechanics is critical when injuries occur from overuse, trauma, or degeneration. Factors such as nutrition, exercise, and many others play an important role in the health of the musculoskeletal system. Understanding the mechanisms of these relationships provides multiple targets for minimizing injury and recovery. The primary purpose of this investigation is to understand how microbiota may affect the physiological processes that are essential for survival. At the same time, it also aims to understand how dysfunctional musculoskeletal systems could affect the microbiota. From there, the interactions can begin to be dissected, and potential therapeutic targets can be identified [10].

## 2. Interactions Between Microbiota and Musculoskeletal System

The musculoskeletal system and microbiota have a complex and reciprocal connection. The microbiota has an effect on the musculoskeletal system, and the musculoskeletal system has an impact on the microbiota. Interaction between the musculoskeletal system and microbiota is facilitated in a bidirectional pathway. Muscle and bone tissue are influenced by the composition of the microbiota. Several mechanisms can illustrate how microbiota can affect musculoskeletal health. Metabolites and signaling molecules are secreted by microbiota that can have pro-inflammatory, anti-inflammatory, or immunostimulant effects, modulating low-grade systemic inflammation, which is associated with various musculoskeletal conditions. The gut–brain axis can influence the musculoskeletal system and vice versa. Research has shown evidence of a reciprocal communication connection between the gut and the brain. Furthermore, systemic inflammation can diffuse from the gut to other parts of the body and affect musculoskeletal injury [2]. Understanding the mechanisms underlying the healthy microbiota–musculoskeletal system is important in recognizing the effect of dysbiosis on musculoskeletal conditions. The chronic health burden is represented by the rise in musculoskeletal injuries. With the aging population, the prevalence of musculoskeletal injuries is expected to increase. This makes it important to have a healthier lifestyle. Novel and non-invasive therapies for prevention and treatment manipulate and modulate the microbiota’s composition. The implications of these therapies for musculoskeletal conditions are significant because an association between the microbiota and musculoskeletal conditions has been demonstrated by increasing evidence. Possible health benefits and disease prevention will be obtained by promoting healthy microbiota and a balanced musculoskeletal system [11,12].

### 2.1. Mechanisms of Communication

It has been known for a long time that microbes can affect their host—usually considered in the context of infection and disease. In this regard, the comparison of models of microbial exposure to germ-free animal models (which have skinnier bones and are shorter, smaller, and thinner) shows the major role played by the host immune system [2]. Research on how physiologically relevant microbial metabolites—bacterial components, excreted waste, secreted enzymes, etc.—signal to and affect bone tissues through transportation and circulation is in its infancy. Microbial metabolites may influence local gut inflammation, which, if not properly controlled, can significantly contribute to osteoarthritis and related functional impairments. On the flip side, systemic inflammation regulates bone mass and microarchitecture. Intestinal inflammation has also been found to be associated with chondrocyte colony formation in vitro, verifying an arthritic link. This review explores both established and potential communication pathways between microbiota and the musculoskeletal (MSK) system, highlighting microbial metabolites as key but often underappreciated signaling molecules. It also discusses possible interventions to enhance beneficial microbial interactions and the challenges imposed by the complexity and multifactorial nature of these biochemical dialogs. This is a bidirectional interaction, with growing interest in understanding how the microbiota influences the host, particularly through nutrient processing and biological waste management. Beyond the gut, the skin is continuously exposed to diverse microbial signals from both pathogens and commensals, adding another layer to host–microbiota communication. Perturbations to normal communication result in widespread health issues, although these interactions are only beginning to be elucidated. Much work remains to fully reveal the physiological significance and scope of the many pathways and signaling mechanisms involved in the dialog between microbes and the host. The gut microbiota influences host musculoskeletal health through the action of gut-derived metabolites, building the foundation imperative for developing strategies which exploit these mechanisms for health benefits. The biochemical dialogs in the MSK system are initiated directly by bacteria-affecting hormones, growth factors, and vitamins, which can regulate the whole organism’s homeostasis. While discoveries of additional communication pathways remain underappreciated and elusive, there have been significant recent advances linking gut and skin health through microbial action [10,11].

### 2.2. Impact on Inflammation and Immune Responses

The impact of microbiota on the host’s health is unquestionable. For a long time, research has demonstrated numerous biological functions modulated by commensal microbes, ranging from metabolite production to complex immune signaling. Regarding the musculoskeletal system, microbiota also play significant roles in controlling health and physiology. Using direct and indirect effects, an intact symbiosis may keep inflammatory responses under control in numerous parts of the system, inhibiting uncontrolled inflammatory processes. On the other hand, dysbiosis may lead to unsuitable inflammatory patterns, enhancing the damaging of musculoskeletal components. Relevant cytokines are produced by microbiota with direct interaction with the immune system, but probably, the main role of microbiota lies in their ill-defined but clear conjoint ability to trigger or inhibit inflammation [2]. In response to inflammation and autoimmune balance, microbiota are likely to intervene by modulating the overall load of potent inflammatory signals in three ways. Primordial microbiota impairment, per se, is likely a source of immunological disturbance that would propagate chronic inflammation. This would likely solve a gap between a common constatation of musculoskeletal clinical condition exacerbation and the recent understanding of gait dysbiosis. This concept intends to highlight how simple musculoskeletal conditions such as a chronic back pain could trigger alterations in microbiota populations that, in turn, would promote a low level of local inflammation, which, over a long period of time, would increase pain levels and lead those patients to a benign but difficult-to-treat condition of chronic lower back pain [13].

## 3. Microbiota Dysbiosis and Musculoskeletal Injuries

Nearly one-third of the world’s population will experience a non-catastrophic musculoskeletal (MSK) injury at some point in their life. While more than 50% of injured people should expect to recover within the first 6–12 weeks, about 20–25% transition to chronic pain, experiencing a decline in physical activity, impaired mental health, higher healthcare costs, and poor quality of life. However, the etiology of chronic musculoskeletal (MSK) disorders is poorly understood, with currently no effective and seamless clinically transitioned prevention strategies. The latest theoretical models argue for the framework of an exposure-distress pathway initiated by an initiating exposure (IE), such as trauma or infection leading to organic injury and immune responses, with unmodifiable and modifiable factors altering recovery trajectories. Although promising, a number of knowledge gaps in these models continue to hinder the prospect of early risk identification, contributing to early intervention. One potential reason for stalled progress remains the failure to appreciate the interpersonal variance stemming from biological or genetic make-up, lifestyle, behaviors, and environmental conditions influencing the biology of the individual in injury recovery [14,15]. A person’s individual microbiome is accepted to be one of the major contributors to explaining that variance, as suggested by recent work. The collective microbial populations in the environment are defined as the microbiota, with the colon providing refuge to over 100 trillion microorganisms, mostly bacteria. The effect of microbiota on host immunology, digestion, and metabolism has drawn wide attention, as it also plays pivotal roles in the presentation and development of both CNS and peripheral MSK [16]. Flighting the factors of health problems caused by microbiota imbalance, dysbiosis is characterized as more diversity and decreased stability of already-present microbes or by an explosive increase in some otherwise-rare outsiders. Dietary habits, physical activity, lifestyle, history of antibiotic use, and the type of birth have been identified as some factors that might cause an alteration in the natural balance, though it has been reported that the spatial–temporality of microbiota makeup also has a critical role. Dysbiosis exerts its effects from the colon and circulatory system on overall health deterioration, as seen in a large variety of diseases ranging from digestive system disorders to chronic inflammations and almost every neuro-pathological disorder like Alzheimer’s and autism. Originating from the intestines and colon and translocating to blood circulation, pathobionts like Enterococcus Gallinarum and certain Firmicutes and Proteobacteria types have been shown to exacerbate inflammation with hostile metabolites, hijacking the immune system, which is most frequently associated with the musculoskeletal-related injuries reported most explicitly in orthopedic cases. Restoring the microbiota’s diversity and stability following a musculoskeletal injury may catalyze recovery, as recent findings have elucidated gut–brain cycles or pathways which involves HPA axis modulation and consequential nerve transfers. Co-cultivation, metabolite swapping, and physio-chemical effects are other potential strategies that have been subject to very limited research.

### 3.1. Definition and Causes of Dysbiosis

Dysbiosis stems from the Greek words “dys”, meaning bad or disordered, and “bios”, meaning life or living organism, to suggest a condition in which the balance of microbial communities is disturbed. This term was introduced in 1998 to distinguish quantitative changes in the microbiota from qualitative changes in the presence of pathogens. Dysbiosis can manifest in various ways, such as shifts in the proportion of symbiotic and pathogenic bacteria, significant modification of the number of stable microbial communities, or a change in the nature and functions of microbial communities. Even though the balance of the microbiota in a healthy person may fluctuate with various factors, it is often stable. Dysbiosis can be triggered by environmental changes and intrinsic/extrinsic factors such as a sudden change in lifestyle, dietary intake, medication, and xenobiotics. This might lead to alterations in the mutual relationship between the host and the microbiota. As a result, the disruptions within microbial communities can harm the host, causing various diseases [17]. The body of this manuscript focuses on altering the microbiota profile so as to improve musculoskeletal health and treat musculoskeletal injuries. This approach is built on the idea that the microbiota is essentially the key to overall health and, more specifically, that variations in the microbiota can cause the deterioration of musculoskeletal health while restoring microbial balance. This offers a promising way forward in protection against such conditions. By reviewing this paper, publications and re-analyzed data from a major musculoskeletal study can provide clear evidence that microbiota-wide imbalances are linked to musculoskeletal health and injuries, thereby informing potential intervention strategies and opening a window onto an area which is currently poorly understood. Furthermore, it is conceivable that dysbiosis of the microbiota occurs at different degrees, depending on an individual’s genetics and lifestyle. Given the need for personalized analysis and intervention, the degree of a muscle injury in relation to dysbiosis should be analyzed with respect to protected genetic and lifestyle characteristics [16]. 

### 3.2. Link to Musculoskeletal Injuries

Understanding basic science principles such as “for every action there is a reaction” is essential for comprehending the general interactions within the body and the potential relationships that the physiologic environments may have with one another. Currently, the “outside” environment being referred to is the gut microbiota, and how it may be linked to musculoskeletal injury will be examined. Musculoskeletal injuries affect the bones, muscles, ligaments, tendons, and nerves in the human body. This section of research will restrict the link to microbiota dysbiosis and the inflammatory response to an injury. This approach may seem somewhat niche compared to the broad spectrum of how the gut microbiota could be entangled within injury; however, when viewing the inflammatory process following an injury, this untrodden scientific interaction of within-body environments may entice fresh horizons [18,19,20].

**Figure 1:**
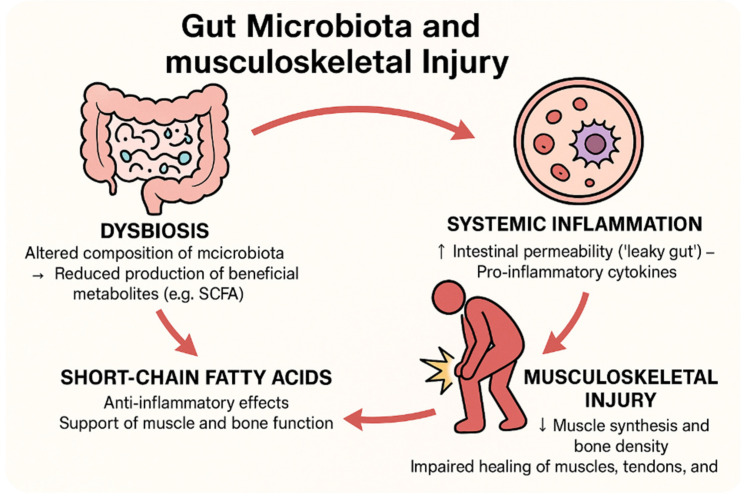
Interactions between gut microbiota and musculoskeletal injuries.

Inflammation is the fundamental bodily response to injury, resulting in the accumulation of blood and cellular content at the site, in hopes to initiate the start of the healing process. Low-grade inflammation can be beneficial after an injury, but sustained chronic inflammation will exacerbate any damage and diminish the healing process. In particular, it is our intention to examine inflammation and the following musculoskeletal conditions: after an impact injury; the sprouting of osteoarthritis (affecting the bone and possibly the surrounding mucosa); following a fracture (this could be caused by impact or a separate environment-dependent condition); and damage to a tendon (which connects a portion of the muscle to the bone and is not directly innervated). Carefully selected examples of the latest findings related to the microbiota that could exacerbate the inflammation process following these injuries will be noted as well. With the emergence of a new gut–brain communication axis, research into how the gut microbiota can influence other bodily environments is rapidly expanding in the hopes of new therapies [16]. Research has already tried the simple colonization of good microbes via novel clinical trials to further close our knowledge gap regarding the significance of the gut microbiota and the musculoskeletal link; therefore, it is the intended hope of this study to investigate explorations of this relatively new field of research [21]. Below is an image (Figure 1) explaining the interactions analyzed in the previous paragraphs.

## 4. Experimental Models and Studies

Microbiota research represents a multidisciplinary and rapidly evolving field that investigates intricate interactions between commensal and pathogenic microorganisms resident in the human body and the host tissues in terms of health and disease. Recently, possible relationships between microbiota and the musculoskeletal system have been actively discussed. The interest in microbiota research is spreading not only within clinical research but also in various non-clinical but essential studies such as mental health and behavioral science. Basic microbiota studies use various experimental models and methodologies, such as host–pathogen studies and pathophysiological studies by the artificial administration of selected bacteria or chemicals. One key figure in these basic studies was Robert Koch, who made Koch’s postulate a standard for establishing the causal relationship between an organism and a disease. This postulate (a) provides a single agent, (b) establishes pure culture of the agent and examines the disease caused by the administration of the pure culture organisms in animals, and (c) reisolates the agent from the animals. The above evidence demonstrates the significance of in vivo and in vitro models to address the complex interaction and functionality of complex microbiota. Many experimental models have been used in various biomedical studies to suggest possible relationships and outcomes regarding musculoskeletal injury and microbiota, due to the two-way communication between the gut and skeletal muscle in terms of inflammation, metabolites, and cytokines. Traditional studies are usually based on animal or in vitro model's microbiota analysis in terms of types and abundance of microbiota using tools of microbiology, molecular biology, and bioinformatics. The starting point of this review was the limitations in microbiota research and musculoskeletal injury studies as preliminary experiments to investigate the feasibility study. This study is based on a literature search and in-depth analysis [19].

The mechanisms of musculoskeletal injuries, the role of the microbiota, and the related effects that have been analyzed in the previous chapters are reported in Table 1, with the related sections in which they are described in detail.

### 4.1. In Vivo and In Vitro Models

This paper reviews the emerging body of literature investigating the associations between the microbiota and various aspects of musculoskeletal pain, including pathogenesis, diagnosis, prognosis, treatment, and management. There are some overarching common themes within the literature, as well as connections to the brain–gut axis and immune activation pathways. The review follows a narrative structure to discuss the gut microbiota, oral microbiota, and dysbiosis in general. Given the rapid rate at which the field is developing, guidelines for future research efforts are suggested. This review paper describes examples of existing research related to connections between the microbiota and the genesis, crosstalk, diagnosis, prognosis, prevention, and treatment of musculoskeletal pain. A primary focus is on the various types of musculoskeletal pain emanating from the neck to the legs, including spinal pain and extremity complaints in between. In vivo models include mice, rats, zebrafish, and more. In vivo models span from specific germ-free conditions to complex community, natural, and synthetic dysbiosis, alongside a broad spectrum of musculoskeletal studies, including arthritis of several forms, osteoporosis, muscle injury and regeneration, development, and engineering. Examples of recent studies show novel findings that mold the critical importance of the microbiota for musculoskeletal health in vivo [10,22].

### 4.2. Key Findings from Research Studies

New paradigms of health and disease are continuously evolving with innovative approaches in medical research. Based on this evidence, contemporary scientific and clinical research could disclose the central role of an unexpected actor, the microbiota, in the health and disease of apparently unrelated body parts. After conducting an extensive narrative review of the current literature, the aim of this investigation is to specifically address the associations between the gut microbiota and musculoskeletal health and pain [2]. The outcomes will aid researchers and clinicians to further understand specific basic scientific and clinical settings, fulfilling academic research needs and designing a tailored pertinent healthcare solution. Numerous pivotal peer-reviewed studies have been conducted, which unravel how the composition of the microbiota, governed by genetics, environment, and stochasticity, can impact health and disease in different areas of the body, with a particular focus on musculoskeletal involvement.

## 5. Clinical Implications and Therapeutic Interventions

Microbiota research delivers a growing understanding of the microbiota’s diversity, complexity, and influence on the physiology of different organs and bodily systems. The impact of the microbiota on the musculoskeletal system is starting to draw attention in the research community but so far is understudied. Ongoing efforts include basic research studies in arthritic diseases or joint-related issues, adverse effects of fitness training regimens, injuries, and inflammation. As microbiotic zones are in close contact with the different musculoskeletal structures, a visible impact of the microbiota on orthopedic and trauma-surgery settings can be suggested. There are several clinical reports about the microbiota profiles of patients contracting infections or experiencing delayed healing and recurrent issues even after successful operations. Although the reasons and the specific roles of the microbiota in these aseptic-setting complications are not yet fully understood, they may hide valuable knowledge for the construction of an advanced perspective, prevention, or treatment strategy. Microbiota research can help clarify the underlying causes of complications, even in less-common post-surgical issues such as surgical site infections, septic arthritis after arthroscopic interventions, non-union healing after fractures, post-amputation phantom pain, and complex regional pain syndrome type I. Additionally, it may shed light on the recurrent appearance of herniation or displacement of operated disks, as well as the appearance of muscle injuries even after seemingly successful healing. This overview may help draw attention to funding bodies for more specific and potentially productive research plans. With the collaboration of basic researcher groups, this initiative review stands as a theoretical base to stimulate clinician researchers to achieve novel results. Establishing the concurrence of the challenges elucidated in this paper may also boost the advent of innovative ideas, leading to publication in reputable, widely read scientific journals [23,24,25]. 

### 5.1. Diagnostic Approaches

This collaboration aims to explore a futuristic model of trauma care—one which converges bioinformatics with minimally invasive health monitoring to tailor psycho-bio-behavioral interventions for recovering motion after trauma. There are plans to adapt the model-centric approach for recovery childhood trauma in the future. The focus will be on the models developed in spinal injury and spinal micro-fontanel anatomy and pointing out how computer-aided techniques might improve clinical outcomes.

### 5.2. Potential Therapies and Interventions

Emerging evidence supports the role of the gut microbiota in musculoskeletal health. Compelling studies demonstrate that the health of the gut microbiota directly influences outcomes after musculoskeletal injury. Indeed, dysbiosis has consistently been associated with a multitude of diseases, including musculoskeletal injuries. The restoration of the microbiota has been associated with mitigated inflammation and improved recovery after musculoskeletal injury [19]. Multiple therapies targeting the microbiota are being investigated, which could be implemented to promote musculoskeletal health. For instance, microbiota disruptions can be mitigated through a variety of interventions, including non-invasive ones such as probiotics, prebiotics, and dietary modifications, which have the potential to restore microbial balance. Many trials are or have been conducted with an orthopedic and musculoskeletal focus, investigating therapies targeting the microbiota, with promising results as a basis for future works. The translation of interventions targeting the microbiota into clinical practice is complicated by the highly individualized nature of microbial communities. Two people may respond differently to the same therapy or display a similar outcome in response to different therapies. However, the broad exploration of therapies and the mechanistic understanding of musculoskeletal injury are establishing the microbiota as a therapeutic target, pushing toward the adoption of a microbiota-centric treatment approach in the near future. Future studies pairing a personalized treatment plan for musculoskeletal injury with a tailored therapy targeting the microbiota hold promise for significant developments in patient care [26,27].

## 6. Future Directions and Research Opportunities

Alterations in the natural microbiota, conventionally named dysbiosis, play a fundamental role in maintaining health or promoting disease states. Since the microbial inhabitants regulate a large extent of the host genome, it is conceivable that they have the potential to affect health conditions far beyond gut maladies, interact with the immune system, and modulate the host’s risk for orthopedic and musculoskeletal injuries [2]. In this context, microbiota research will have to develop in more innovative ways, focusing specifically on the influence of exogenous factors—such as diet, immune-compromising or essential drugs, unexpected frailty and climate and other environmental determinants—enlarging the scale course of studies on this topic. Areas of interest for future research include the potential effects of anti-inflammatory nutrients on the microbiota’s composition and association with musculoskeletal injuries or the application of artificial intelligence and machine learning to large amounts of omics data. Musculoskeletal diseases are the most prevalent medical condition affecting populations in both developed and developing countries, causing pain and disability in the workplace. However, biomedical advancements in musculoskeletal research and treatment have mostly concentrated on the joints and the nearby tissues, underestimating the systemic and complex interplay of pathogens, genetic factors, and environmental and lifestyle conditions. The gut, where more than 70% of the human immune cells are based and where at least half of all blood proteins are synthesized and metabolized, has been a neglected topic. Yet, the gut presents an exceptional microbial habitat that has recently gained strong interest thanks to its many implications, including in protein synthesis, metabolite elevation, protection against potential pathogens, and epithelial differentiation. Moreover, due to the deep phylogenetic distance between humans and bacterial pathogens, commensal inhabitants are technically safe houses from bacterial infections, explaining the wide employment of probiotic bacteria [19,28,29].

### 6.1. Emerging Areas of Study

Throughout the field of medicine, there is an increasing appreciation for the importance of the human microbiota. While, historically, the musculoskeletal system has been studied as an isolated organ system, this system is now understood as interacting with numerous other organs as part of a singular, comprehensive organism. Several questions arise in exploring the microbiota and its potential impact on musculoskeletal health, in addition to a consideration of the types of microbiomes found in the gut or on the skin. These include how the microbiome changes with age, diet, or activity and when the same person is healthy vs. when they have musculoskeletal pain [2].

Within the framework of these questions, there are a number of noteworthy emerging areas of study. The first involves how exercise influences the microbiota and vice versa. Next, there is interest in the dietary impacts on the microbiome and musculoskeletal health. Additionally, an understanding of the microbiome’s involvement in chronic pain syndromes and musculoskeletal conditions is sought. Furthermore, there is pioneering research into the gut biome’s involvement in recovery from musculoskeletal injuries. These areas of multidisciplinary research provide a synopsis of the next steps in exploring how the microbiome interacts with the musculoskeletal system. Additionally, studying the gut–brain axis may provide unique insight into aspects of musculoskeletal health such as overtraining, development of fibromyalgia following trauma, or chronicity of lower back pain [16]. An overview of practical implications for musculoskeletal health may also provide a direction for progress in the field toward optimally treating these conditions. Importantly, several of these needs are theorized to overlap with potential new therapeutic targets of the microbiome to treat musculoskeletal conditions. Emerging musculoskeletal topics relevant to a microbiota-based inquiry have also been discussed in this paper, as an invitation for researchers to expand their understanding to broader aspects of the interplay between musculoskeletal conditions and different microbiomes.

### 6.2. Technological Advancements

The recentness of human microbial science has had both propellant and inhibitory effects on the field. While this is an exciting time for discovery and innovation, scientists and other stakeholders not directly involved in this research can feel overwhelmed or unsure of the possible implications for real-world applications. This review has attempted to consolidate the more recent origins of the microbiota–musculoskeletal association and envision near-term advances in microbiota-focused musculoskeletal research and links to telemedicine, digital health technologies, and other newer technologically driven facets of medical care. Groundbreaking developments in our comprehension of the human microbiota are now underpinning changes in how bone and joint injuries, sports, and occupation-related conditions are perceived. Since its conception in 2008, research on microbiota has rapidly expanded, but there are relatively few reports on the musculoskeletal envelope. The unearthing of the microbiota’s influence on the workings of the immune system, metabolism, and host genetic expression is providing a new interpretative grid for musculoskeletal ailments. New animal microbiota iteration-based models are portraying how changes in gastric and peripheral microbial colonies can influence bone and joint health. Similarly, the microbiota’s influence on muscle architecture and functional performance can affect joint strain and show how certain microbial shifts can circumvent musculoskeletal injuries [30]. Given all the recent developments and current state of microbiota-focused technology in musculoskeletal and other fields, establishing near-term advancements and hypotheses concerning microbiota-focused bone health studies is of interest to a wider audience. In addition to being an innovative period for musculoskeletal biology and microbiota research, it is proposed that this is a moment of transition in how personal health management occurs. Policymaking and sponsorship of recent medical advancements, particularly those focusing on, or tied to, variously connected databases, can assist in envisaging future engagements with microbiota-associated musculoskeletal anomalies. The bacterial attacks that could potentially avert musculoskeletal injuries are only rudimentarily understood. In tandem with the explosion of combinatorial drugs and gene manipulations, pharmaceutical modification can be viewed contractually as part of an all-around plan for faster, more pointed orthotic and healing interventions in the future [31].

## 7. Conclusions and Summary

For many years, the idea that keeping one’s body in good health was a duty, the failure of which would make one unable to keep their mind strong and clear, guided philosophers, politicians, and thinkers alike. These words also have significant scientific implications. Recently, a groundswell of research has underlined the ties between the microbiota and the mechanisms involved in the development of osteoarthritis (OA) and musculoskeletal injuries. Musculoskeletal health and the prevention and treatment of musculoskeletal injuries have long been a distant, niche concern from the point of view of the health of the wider public. However, some of these worldwide health issues might serve as a signal in favor of broadening concern for musculoskeletal health to potential investors in population health. However, focusing on the gut microbiota, a significant link between nutrition and disease, may develop a strong area of interest, closely engaging musculoskeletal health [2]. Microbial dysbiosis has been associated with many pathological conditions, such as non-alcoholic fatty liver disease, hypertension, inflammatory bowel disease, immunosuppression, and various types of cancer. In addition, it can also increase the risk of injuries. However, a significant number of diseases and dysfunctions remain unexplored, and it is likely that many bacterial species are still to be discovered. Nowadays, the idea that controlled attacks on these gut bacteria could treat pathology is opening new horizons in so-called precision medicine. Several strategies have been designed to enable us to gain deep insights into the correlation between the microbiota, musculoskeletal injuries, and health, as well as figure out new effective methods of prevention, diagnosis, and treatment [16]. This cascade of progressive damage can trigger an inflammatory response, ultimately leading to chronic pain and osteoarthritis. Increasing evidence has pointed out that gut microbiota are also involved in chronic pain development, implying that antibiotics could be used in chronic pain treatment. This study sheds light on the physiological and pathological interactions between the gut microbiota and musculoskeletal injuries, which can benefit the investigation and management of the latter. Furthermore, alternative therapies such as dietary interventions and supplements have become available, based on the ways in which the microbiota can be modified. As a result, there has been a recent, usual increase in interest related to study of the mysterious human microbiota. Musculoskeletal injuries affect almost everyone at some point in their life, either as the natural result of the human body aging or because of sudden traumas. Advances in scientific research, new preventative measures, and efficient protective strategies have improved people’s health and life expectancy, consequently leading to most industrialized countries undergoing drastic cultural and demographic changes. The novel organized interaction between biomechanical approaches, nutrition aspects, surrounding cultural and geographical variations, and eventual effect on the microbiota opens a new landscape for further advances in investigation and prevention. Musculoskeletal injuries are not only a side pathology or consequence of other health issues but rather the result of numerous interconnected processes, which require a multidisciplinary approach in order to be fully understood. Many functions of the human body are controlled daily by its own microbiota; most of the attention so far has been given to the gut–skin–lung axis, even though its role in the musculoskeletal system still remains a mystery. While displaying high biomechanical performance, musculoskeletal tissues rely on complex metabolic cycles, which might be connected to the microbiota. Finally, a possible effect of diet-induced changes in the gut microbiota on skeleton and muscle performance should be considered, thus opening a new prospective branch of research on musculoskeletal injuries [18,21].

## Figures and Tables

**Table 1:** Summary table of key mechanisms in the microbiota–musculoskeletal axis.

Mechanism	Role in the Microbiota–Musculoskeletal Axis	Effects on the Musculoskeletal System	Reference in the Article
Microbial Metabolites (SCFA, LPS, TMAO)	Regulate inflammation, bone metabolism, and muscle function	Improve bone density and reduce chronic inflammation	Section 2.1, Mechanisms of Communication
Systemic Inflammation	Pro/anti-inflammatory cytokines (TNF-α, IL-6, IL-10) influence bone turnover	Modulates balance between bone formation and resorption	Section 2.2, Impact on Inflammation and Immune Responses
Immune System	Modulates inflammatory response and tissue repair	Promotes injury recovery and reduces muscle damage	Section 3.2, Link to Musculoskeletal Injuries
Gut–Muscle Axis	Connection between microbiota, energy metabolism, and muscle function	Affects muscle strength and sarcopenia risk	Section 3, Microbiota Dysbiosis and Musculoskeletal Injuries
Intestinal Dysbiosis	Altered microbiota composition affects inflammation and oxidative stress	Increases risk of osteoporosis, arthritis, and muscle damage	Section 4, Experimental Models and Studies

## Data Availability

Not applicable.
